# Effect of Industrial Solid Waste as Fillers on the Rheology and Surface Free Energy of Asphalt Mastic

**DOI:** 10.3390/ma17051125

**Published:** 2024-02-29

**Authors:** Li Ou, Hongzhou Zhu, Ruipu Chen, Chunli Su, Xiaosi Yang

**Affiliations:** 1 School of Civil Engineering, Chongqing Jiaotong University, Chongqing 400074, China; ouliyx@126.com (L.O.);; 2 National & Local Joint Engineering Research Center of Transportation and Civil Engineering Materials, Chongqing 400074, China; 3 School of Transportation and Civil Engineering, Shandong Jiaotong University, Jinan 250357, China; 4 CCCC Second Highway Consultants Co., Ltd., Wuhan 430056, China

**Keywords:** asphalt mastic, solid waste, rheology, surface free energy

## Abstract

The continuous growth of industrial solid waste production has generated many environmental problems. We evaluated the potential of industrial solid waste as a substitute filler in asphalt mastic, with the aim of increasing the use of sustainable road construction materials. In this study, X-ray fluorescence spectroscopy (XRF) and scanning electron microscopy (SEM) were used to characterize the oxide composition and micromorphology of limestone (LS), red mud (RM), steel slag (SS), and ground granulated blast-furnace slag (GGBFS). Four asphalt mastics containing LS, RM, SS, and GGBFS with a filler-to-binder weight ratio of one were prepared. An evaluation of the rheology and wetting of the solid-waste-filler asphalt mastic was conducted using a frequency sweep, temperature sweep, linear amplitude sweep (LAS), multiple stress creep and recovery (MSCR), and surface free energy (SFE) methods. The results showed that SS increased the complex modulus, elastic component of the asphalt mastic and decreased the nonrecoverable creep compliance at stress levels of 0.1 and 3.2 kPa, which improved the rutting resistance of the asphalt mastic and reduced deformation under high-temperature conditions. The RM and GGBFS increased the fatigue performance of the asphalt mastic under strain loading, enhanced its fatigue life, and maintained good performance under long-term loading. The dispersive component of the SFE parameter of the solid-waste-filler asphalt mastic was larger than the polar component for the largest share of the surface energy composition. The SFE of the asphalt mastic prepared from the industrial solid-waste filler was reduced; however, the difference was insignificant compared to the limestone asphalt mastic. Solid-waste-filler asphalt mastic has performance characteristics, and its actual application can be based on different performance characteristics to select an appropriate solid-waste filler. The results of this study provide new technological solutions for solving the utilization rate of solid waste materials and sustainable road construction in the future.

## 1. Introduction

Asphalt pavements have become the preferred structure for new pavements because of their ideal driving comfort, continuity, and traffic early after paving [1]. The performance of asphalt pavements is influenced by multiple factors. External factors, such as temperature, humidity, and traffic loads, increase the complexity of asphalt pavement stresses [2]. Asphalt concrete is a multiscale composite material comprising asphalt, fillers, and aggregate [3]. Asphalt mastic comprises asphalt and filler, and its performance is correlated with the pavement performance of asphalt concrete [4]. The physical and chemical properties, morphology, mineral composition, particle size, surface texture, and other factors of fillers can affect the performance of asphalt mastic [5]. Fillers can enhance the high-temperature resistance to the rutting deformation, fatigue performance, and water damage ability of asphalt mixtures and effectively reduce porosity [6,7,8]. When the filler enters the voids of the asphalt mixture, it serves as an additional contact point between aggregates, providing better stress distribution and effectively dispersing stress when subjected to loads, avoiding stress concentrations [9].

Asphalt mixtures require the extensive exploitation of natural resources, which has an irreversible destructive effect on the environment [10]. As the problem of environmental pollution becomes increasingly prominent, reducing it and improving the environment are of significant importance to human life. The concept of environmental protection has become popular globally, and countries are gradually establishing and adopting targeted environmental governance measures to protect and improve the environment and meet the needs of sustainable development strategies [11]. With the intensification of urbanization, large amounts of industrial solid waste are produced. Industrial solid waste has the dual attributes of both pollutants and resources [12]. Industrial solid waste replaces some raw materials and helps reduce carbon emissions [13]. The recycling of solid waste materials is consistent with sustainable development, and solid waste powders can replace limestone (LS) filler while improving the performance of asphalt concrete. Many researchers have analyzed the behavior of asphalt mixtures containing waste as fillers. Various waste materials, including phosphogypsum [14], zinc production waste [10], ceramic waste [15], Ground-granulated blast-furnace slag (GGBFS) [16], Red mud (RM) [17], and Steel slag (SS) [18], have been added to asphalt mixtures. The use of solid-waste fillers in asphalt enhances the absorption of asphalt and reduces waste landfills [19].

GGBFS is the waste slag discharged from a blast furnace in a molten state during the smelting of pig iron. It is quenched and cooled sharply by water quenching, and most of it is glassy with potential water-hard gelling properties [20]. GGBFS can be used as a substitute for cement, resulting in significant savings in construction costs [21]. The performance of the mixture formed by adding GGBS in a cold asphalt emulsion is significantly improved compared with that of a hot asphalt mixture [22]. The performance of the hot mix asphalt mixture was evaluated by adding different amounts of GGBFS, and the results showed that the asphalt mixture with 3% GGBFS had a good fatigue life and anti-rutting ability [16]. However, GGBFS mainly focuses on the research of the cement industry, cold asphalt emulsion, and asphalt mixture, and little research has been found on the scale of asphalt mastic as a filler.

RM is a solid waste produced during the extraction of Al_2_O_3_ from bauxite ore and has a reddish appearance owing to its high Fe_2_O_3_ content [23]. RM is divided into three methods according to the different aluminum production processes: Bayer, sintering, and combined methods [24]. RM is used to extract rare earth elements and prepare environmental remediation materials and cementitious materials [25,26]. The sintering RM asphalt mastic has a larger complex modulus ($G^{*}$) and elastic recovery than the Bayer RM asphalt mastic, the cumulative creep strain is smaller, and the improvement in the high-temperature performance of the Sintering RM asphalt mastic is more significant [17]. Compared with the traditional LS asphalt mixture, the RM asphalt mixture exhibits higher tensile strength [27], while the water damage resistance performance of the RM asphalt mixture has decreased [28]. Researchers mainly focus on the impact of RM on the performance of asphalt mixtures and evaluate the performance of asphalt mastic through rheological methods. There is little research on the surface free energy (SFE) of RM asphalt mastic.

SS is the molten slag discharged during the steelmaking process and is mainly derived from oxides formed after the oxidation of silicon, calcium, and iron [29]. SS has good conductivity and can be used as the aggregate in asphalt mixtures, and the self-healing performance of asphalt can be improved through microwave heating [30]. The free CaO and MgO phases in SS lead to volume expansion, resulting in poor stability [31]. The inhomogeneity and poor stability of SS is drastically reduced by grinding, making it a replaceable filler in asphalt mastic [32]. SS powder asphalt mastic has better aging resistance than LS, which is true for salt and alkaline solutions [18]. Replacing fillers in asphalt mixtures with SS powder has better resistance to water damage, permanent deformation, and low-temperature cracking [32]. Due to the excellent electrical conductivity, there have been many studies on steel slag as an aggregate and filler in asphalt mixtures. However, there is relatively little research on the SFE of asphalt mastics.

At present, research mainly focuses on using rheology to evaluate the performance of fillers when combined with asphalt, while there has been little research on the SFE of SS, RM, and GGBS asphalt mastics. For these types of solid waste materials, researchers did not use the same experimental methods for research and used different filler-to-binder weight ratios, so the results of various studies on solid waste materials cannot be compared. The innovation of this study is to use the same experimental methods to evaluate the rheological properties and the SFE of solid-waste-filler asphalt mastic. Considering the environmental friendliness and economic feasibility of solid-waste fillers, this study investigated the feasibility of using RM, SS, and GGBFS as substitutes for LS. 

## 2. Materials and Methods

**Figure 1:**
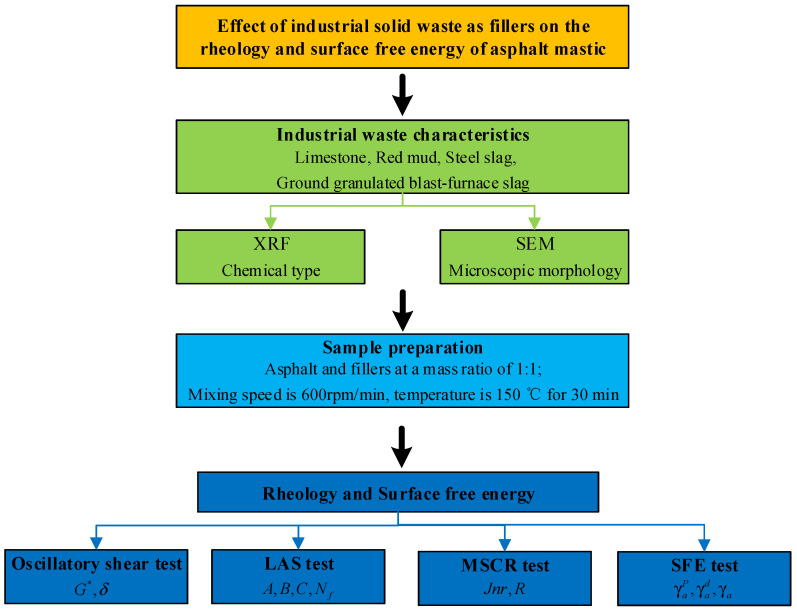
Research program of the study.

In this study, we used three industrial solid waste materials as alternative fillers, RM, GGBFS, and SS powder, to prepare a solid-waste-filler asphalt mastic and compared it with conventional LS powder asphalt mastic. The chemical type and microscopic morphology of the solid-waste fillers were determined by X-ray fluorescence spectroscopy and scanning electron microscopy. The rheological behavior of the asphalt mastic was determined by frequency sweep, temperature sweep, linear amplitude sweep (LAS), and multiple stress creep and recovery (MSCR), and the surface free energy (SFE) test was used to determine the wetting properties of the asphalt mastic. The research program used in this study is shown in Figure 1.

### 2.1. Asphalt Binders

In this study, a pen-grade 60/80 base asphalt binder was used to prepare the asphalt mastic, and the basic properties of the asphalt were tested according to JTG E20-2011 [33]. The test results, which meet the requirements of JTG F40-2004 [34], are shown in Table 1.

### 2.2. Industrial Solid Waste

**Figure 2:**
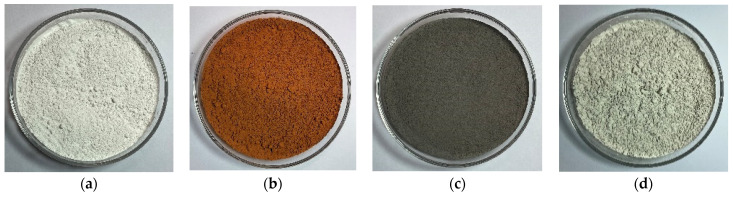
Morphology of four types of fillers: (**a**) LS; (**b**) RM; (**c**) SS; (**d**) GGBFS.

**Figure 3:**
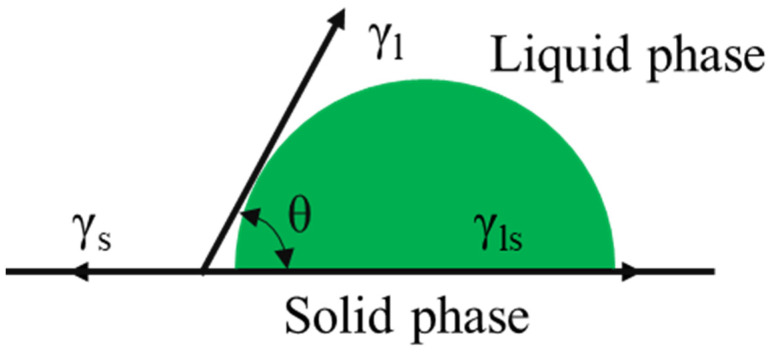
Solid–liquid phase interface contact angle model.

The industrial solid wastes used in this study included RM, SS, and GGBFS. RM was collected from Aluminum Corporation of China Limited. SS, GGBFS, and LS were all obtained from Yuanheng Water Purification Materials Factory in Gongyi City, China. RM, SS, and GGBFS waste powders were compared with the LS powder, and the four fillers are shown in Figure 2. The oxide compositions of the four fillers (listed in Table 2) were analyzed using ARLAdvantX Intellipower 3600 X-ray fluorescence (Thermo Fisher, Waltham, MA, USA). The micromorphologies of the fillers were characterized using a Sigma 500 SEM (ZEISS, Jena, Germany). The particle size analysis of the filler was conducted using the Mastersizer 2000 (Malvern, UK). Owing to the nonconductive nature of the inorganic filler, the surface of the filler was sprayed with gold before the SEM test. The microscopic morphologies of the four filler particles are shown in Figure 3; the four fillers exhibited differences in particle morphology and surface texture.

### 2.3. Preparation of Asphalt Mastic

Asphalt mastic is prepared from asphalt and filler, with asphalt as a dispersion medium and filler as a dispersed phase. To study the effect of the industrial solid-waste filler type on asphalt mastics, asphalt mastic samples were prepared using different fillers. The filler-to-binder weight ratio of conventional asphalt mastic is controlled between 0.6 and 1.4 [35]. In this study, a median of 1.0 was taken to prepare asphalt mastic samples. Before the preparation of asphalt mastic, the fillers were sieved through 0.075 mm and placed in an electric oven at 160 °C for a 24 h drying process to remove moisture from the filler to a constant weight. The base asphalt was heated to 150 °C to make it flowable and mixed immediately to prevent excessive heating from causing asphalt aging. Subsequently, a filler of equal mass to the asphalt was gradually mixed and stirred using a metal bar to prepare the initial mixture of asphalt and filler [14]. Next, the mixture was stirred at 600 rpm for 30 min to ensure homogeneous mixing and no more bubbles appeared on the surface of the mastic. Finally, the homogeneously dispersed asphalt mastics were cooled for subsequent testing.

### 2.4. Rheological Test

#### 2.4.1. Oscillatory Shear Test

The rheological behavior of industrial solid waste asphalt mastic was characterized using a TA DHR-2 dynamic shear rheometer. Frequency and temperature sweep tests were performed on solid-waste-filler asphalt mastic to analyze the viscoelasticity of asphalt mastic in a wide frequency and temperature range. The strain parameters were determined using an amplitude sweep of 0.1%. The temperature sweep test frequency was 10 rad/s, and the temperature range was 20~80 °C at 10 °C intervals.

#### 2.4.2. Linear Amplitude Sweep (LAS) Test

According to AASHTO TP101 [36], the LAS test was conducted using a dynamic shear rheometer with a test temperature of 25 °C. The samples were subjected to short-term aging, rolling thin film oven at 163 °C preheating for 16 h, adjusting the airflow rate of 4000 mL/min, filling with asphalt mastic containing the sample bottle into the ring-shaped metal frame, rotating at a speed of 15 r/min for 85 min. The LAS test has two sweep steps: first, 12 specific frequency sweeps (Hz) need to be performed on the samples at 0.1% strain: 0.2, 0.4, 0.6, 0.8, 1.0, 2.0, 4.0, 6.0, 8.0, 10, 20, and 30 Hz; second, the samples were at a 10 Hz frequency for the amplitude sweep. The parameters α, B, and A and the fatigue life $N_{f}$ of the solid-waste-filler asphalt mastic in the LAS test were calculated using equations [36]. The equations are solved by frequency sweep using Equations (1)–(3).

(1) $$G^{\prime }\left(f\right)=\left|G^{*}\right|\left(f\right)\times \cos\delta \left(f\right)$$

(2) $$\log G^{\prime }\left(f\right)=m\left(\log f\right)+b$$

(3) α=1/m

where $G^{\prime }\left(f\right)$ is the energy storage modulus, MPa; $\left|G^{*}\right|\left(f\right)$ is the complex shear modulus, MPa; δ(f) is the phase angle, °; f is the frequency, Hz; and α is the rheological characteristic parameter.

The cumulative damage of the samples is calculated using Equations (4) and (5).

(4) $$D\left(t\right)≅\overset{N}{\underset{i=1}{\sum }}{\left[\pi \gamma_{0}^{2}\left(C_{i-1}-C_{i}\right)\right]}^{\frac{\alpha }{1+\alpha }}{\left(t_{i}-t_{i-1}\right)}^{\frac{1}{1+\alpha }}$$

(5) $$C\left(t\right)=\frac{\left|G^{*}\right|\left(t\right)}{{\left|G^{*}\right|}_{initial}}$$

where $\left|G^{*}\right|\left(t\right)$ is the time-dependent complex shear modulus in the amplitude sweep test, MPa; ${\left|G^{*}\right|}_{initial}$ is the initial complex shear modulus, MPa; $\gamma_{0}$ is the strain amplitude, %; and t is the test time, s.

C(t) and D(t) satisfy Equation (6).

(6) $$C\left(t\right)=C_{0}-C_{1}{\left(D\right)}^{C_{2}}$$

where $C_{0}$ = 1, $C_{1}$, and $C_{2}$ are obtained by the linear fitting of Equation (7).

(7) $$\log\left(C_{0}-C\left(t\right)\right)=\log\left(C_{1}\right)+C_{2}\cdot \log\left(D\left(t\right)\right)$$

The formula for $D_{f}$ at destruction is shown in Equation (8).

(8) Df=(C0−CPeak StressC1)1C2

The fatigue model parameters A and B are shown in Equations (9) and (10).

(9) $$A=\frac{f{\left(D_{f}\right)}^{k}}{k{\left(\pi C_{1}C_{2}\right)}^{\alpha }}$$

(10) B=2α

where f is the frequency, 10 Hz, and $k=1+\left(1-C_{2}\right)\alpha$.

The fatigue equation is obtained as Equation (11).

(11) $$N_{f}=A{\left(\gamma_{max}\right)}^{-B}$$

where $N_{f}$ represents the fatigue life, times, and $\gamma_{max}$ represents the maximum expected binder strain for a given pavement structure, %.

#### 2.4.3. Multiple Stress Creep and Recovery (MSCR) Test

The MSCR test evaluates the high-temperature performance and elastic response of asphalt materials [14]. The asphalt mastic used in the MSCR test is short-term aging of the sample. Two key parameters were obtained from the MSCR test: percent recovery (R) and nonrecoverable creep compliance ($J_{nr}$). R and $J_{nr}$ were calculated for different stress levels based on Equations (12)–(15).

(12) $$R_{0.1}=\frac{\sum_{N=11}^{20}\left[\in_{r}\left(0.1,\;N\right)\right]}{10}$$

(13) $$R_{3.2}=\frac{\sum_{N=1}^{10}\left[\in_{r}\left(3.2,\;\;N\right)\right]}{10}$$

(14) $$J_{nr0.1}=\frac{\sum_{N=11}^{20}\left[J_{nr}\left(0.1,\;N\right)\right]}{10}$$

(15) $$J_{nr3.2}=\frac{\sum_{N=1}^{10}\left[J_{nr}\left(3.2,\;N\right)\right]}{10}$$

where $\in_{r}\left(0.1,\;N\right)$ and $\in_{r}\left(3.2,\;N\right)$ are the percent recovery, %, for the Nth time at stress levels of 0.1 kPa and 3.2 kPa, respectively; and $J_{nr}\left(0.1,N\right)$ and $J_{nr}\left(3.2,N\right)$ are the nonrecoverable creep compliance, %, for the Nth time at stress levels of 0.1 kPa and 3.2 kPa, respectively.

### 2.5. Surface Free Energy (SFE) Test 

The SFE, which characterizes the energy required to separate substances and thus create new interfaces, was used to evaluate the wetting of the solid-waste-filler asphalt mastic. Surface energy consists of nonpolar and polar dispersion components. Young’s equation explains the relationship between the contact angles of the solid and liquid, the principle of which is illustrated in Figure 3.

In Figure 3, θ is the contact angle between the liquid and solid phase, $\gamma_{l}$ is the surface energy of the liquid phase, $\gamma_{s}$ is the surface energy of the solid phase, and $\gamma_{ls}$ is the surface energy between the solid and liquid. Young’s equation can be derived as shown in Equation (16).

(16) $$\gamma_{l}(1+\cos\theta )=2\sqrt{\gamma_{s}^{d}\gamma_{l}^{d}}+2\sqrt{\gamma_{s}^{P}\gamma_{l}^{P}}$$

where $\gamma_{s}^{d}$ and $\gamma_{l}^{d}$ represent the solid-phase and liquid-phase nonpolar dispersion components, respectively, and $\gamma_{s}^{P}$ and $\gamma_{l}^{P}$ represent the solid-phase and liquid-phase polar components, respectively. It is simplified to the form of y=kx+b to determine the surface energy of asphalt materials [37], as shown in Equation (17).

(17) $$\frac{\gamma_{l}\left(1+\cos\theta \right)}{2\sqrt{\gamma_{l}^{d}}}=\sqrt{\gamma_{s}^{P}}\frac{\sqrt{\gamma_{l}^{P}}}{\sqrt{\gamma_{l}^{d}}}+\sqrt{\gamma_{s}^{d}}$$

The magnitudes of the contact angles of the three types of liquids on the asphalt mastic samples were determined using the lay-drop method. According to Equation (17), the square of the intercept of the fitted curve is the nonpolar dispersion component of the asphalt mastic, and the square of the slope of the fitted curve is the polar component of the asphalt mastic sample.

The asphalt mastic samples were wetted using analytically pure ethylene glycol, distilled water, and glycerol, and the concentrations of all three reagents were greater than 99.9%. The total surface energy, nonpolar component, and polar dispersion component of the three types of test reagents are listed in Table 3, and the contact angle was determined by the laying-drop method using a HARKE-SPACXE-type contact angle tester.

Before the test, thin slices of asphalt mastic were prepared for wetting with different reagents. The preparation of asphalt mastic thin sections was conducted on slides, which were preheated in an oven at 60 °C for 30 min, inserted into the preparation vessel, and hung to cool. Due to the high viscosity of the solid-waste-filler asphalt mastic, the asphalt mastic was thick and uneven, and after it was cooled to room temperature, it was placed in self-leveling at 150 °C for 30 min before being used in the lay-drop method of testing.

## 3. Results and Discussion

### 3.1. Characteristics of Fillers

#### 3.1.1. Microscopic Morphology of Fillers

**Figure 4:**
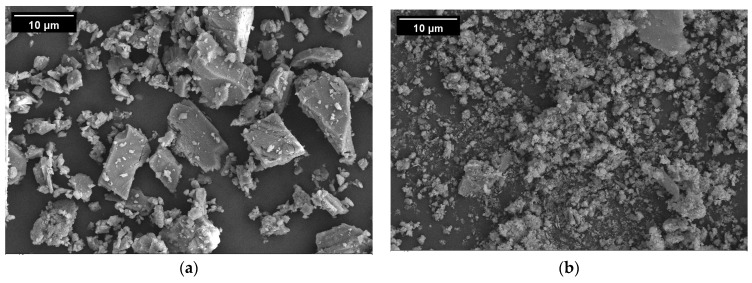

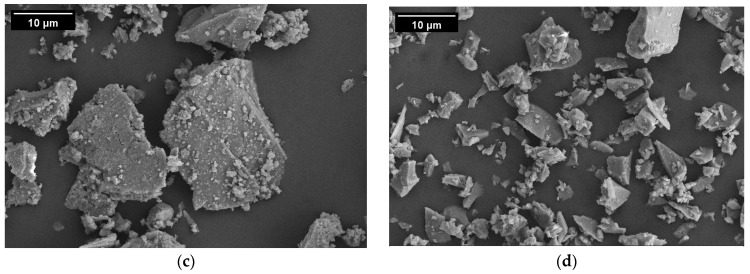
SEM image of four filler particles: (**a**) LS; (**b**) RM; (**c**) SS; (**d**) GGBFS.

Limestone (LS) is the most widely used filler in asphalt mastics, which is compared with solid-waste fillers in this study. The microscopic morphology of LS filler is shown in Figure 4a. The LS filler showed different angular morphologies, clear contours, and polygonal particles with a smooth surface and no holes. The microscopic morphology of the RM is shown in Figure 4b, which indicates that the single particle size of the RM is small and presents an agglomerated particle morphology formed by fine particles. Some of the agglomerated particles are larger than those in the LS powder. The microscopic morphology of RM was the most dense among the four fillers. The microscopic morphology of the SS powder is shown in Figure 4c. It has a rough surface texture, flocculent surface, fluffy structure, developed pores, many holes, and fine channels. Fillers with complex surface textures have high surface activity and can effectively absorb light components and weakly polarized aromatics in asphalt to form an anchoring structure, thereby enhancing the viscosity effect. As shown in Figure 4d, the microscopic morphology of the GGBFS comprises irregular granular, elongated lamellar, sharp-edged, and rough-surfaced particles. The microscopic morphology of GGBFS is affected by grinding processes, such as air milling, ball milling, and vibratory milling.

#### 3.1.2. Chemical Composition of Fillers

From Table 3, the LS powder contains 91.803 wt. % CaO, 3.761 wt.% SiO_2_, 1.727 wt.% Al_2_O_3_, 1.023% wt.% Fe_2_O_3_, and 1.686 wt.% other chemical compositions. The primary chemical composition of the red mud (RM) was 40.245 wt. % Fe_2_O_3_, 26.372 wt.% Al_2_O_3_, 17.009 wt.% SiO_2_, 8.141 wt.% Na_2_O, and 4.260 wt.% TiO_2_. The other oxide components had a total content of 3.793 wt. %. Due to its high iron content, the solid waste is red. Notably, the Na_2_O content in the RM reached 8.141 wt. %, which is at least one order of magnitude larger than the other three fillers. The primary chemical composition of the steel slag (SS) was 30.454 wt. % Fe_2_O_3_, 29.638% wt.% CaO, 21.712% SiO_2_, 6.660% wt.% Al_2_O_3_, 4.337 wt.% MgO, and 3.101 wt.% MnO. The other oxide components have a total content of 4.098 wt. %. The primary chemical composition of the GGBFS was 35.921 wt. % CaO, 31.476 wt.% SiO_2_, 17.176 Al_2_O_3_, 9.810 wt.% MgO, 2.142 wt.% SO_3_, and 1.524 wt.% TiO_2_. The other oxide components had a total content of 1.951 wt. %. Among the four fillers, GGBFS exhibited the highest SiO_2_ content. SiO_2_ is an acidic powdered oxide with complementary properties.

#### 3.1.3. Particle Size Distribution of Fillers

**Figure 5:**
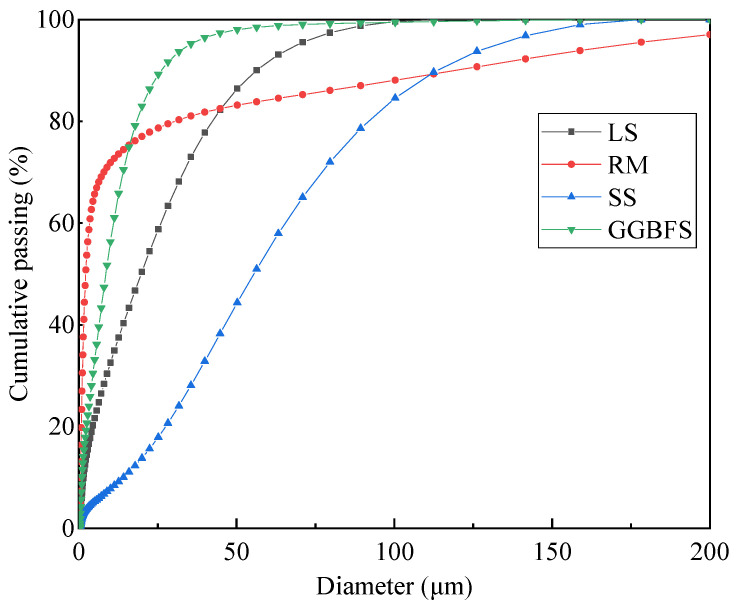
Particle size distribution of fillers.

Figure 5 depicts the cumulative percentage curve of solid-waste fillers to visually analyze the particle size distribution (PSD) characteristics. There are significant differences between the curves, especially within the range of small diameter particle sizes. Red mud has the finest particle size, while steel slag has a coarser particle size, with LS and GGBFS being among them. The particle sizes of LS, RM, SS, and GGBFS with a 50% cumulative passing rate are 19.749, 2.716, 55.435, and 8.539 μm. The particle size may affect the stiffness of asphalt mastic, and the larger the particle size, the more favorable it is for high-temperature performance [14].

### 3.2. Linear Viscoelasticity

#### 3.2.1. Frequency Sweep

**Figure 6:**
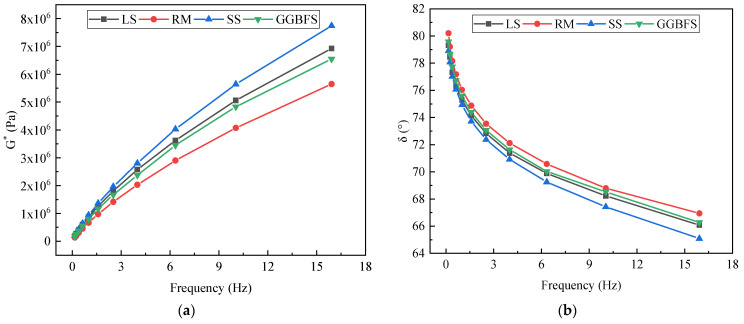
Linear viscoelastic variation of solid-waste-filler asphalt mastic with frequency: (**a**) Complex modulus; (**b**) Phase angel.

Considering the actual driving speed range of pavement vehicles, in this study, we analyzed the viscoelastic properties of asphalt mastic with different solid-waste fillers under the conditions of 30 °C. The $G^{*}$ and δ versus frequency curves for solid-waste-filler asphalt mastic are shown in Figure 6a,b.

From Figure 6a,b, it can be seen that there are differences in the complex shear modulus ($G^{*}$) and phase angle (δ) of solid-waste-filler asphalt mastic. As the frequency of $G^{*}$ increases, indicating that the tire contact with the road surface action time is shorter, the solid-waste-filler asphalt mastic stiffness becomes greater, and the vehicle in the state of high-speed traffic on the road surface damage is smaller. In a frequency sweep, the $G^{*}$ ranking of the solid-waste-filler asphalt mastic was SS > LS > GGBFS > RM. The increase in stiffness is due to better particle disaggregation and a larger filler/asphalt interaction area [38]. The asphalt mastic containing SS had a larger $G^{*}$ value than the asphalt mastic containing LS, indicating better physical stiffening. The enhancement of these effects was mainly because of the chemical composition and particle size of the filler, which led to an increase in the stiffness of the asphalt mastic [39]. The phase angle is an important parameter that reflects the viscoelastic ratio of the internal structure of a solid-waste-filler asphalt mastic. Figure 6b shows the δ variation of the solid-waste-filler asphalt mastic, with δ monotonically decreasing with the increasing loading frequency over the frequency range. Increasing the frequency increases the elastic proportion of the solid-waste-filler asphalt mastic, resulting in a greater elastic response of the asphalt material when subjected to external loading. Using 10 rad/s as an example, the δ-ordering of the asphalt mastic is RM > GGBFS > LS > SS, with RM having the largest proportion of viscous components and SS having the largest proportion of elastic ones. 

#### 3.2.2. Temperature Sweep

**Figure 7:**
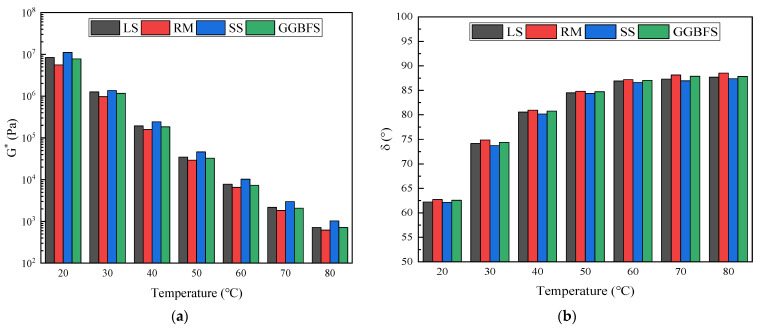
Linear viscoelastic variation of solid-waste-filler asphalt mastic with temperature: (**a**) Complex modulus; (**b**) Phase angel.

Figure 7a,b shows the variation patterns of $G^{*}$ and δ with temperature for different asphalt mastics. Within this temperature range, the $G^{*}$ of SS asphalt mastic is greater than that of LS asphalt mastic, while the $G^{*}$ of GGBFS and RM asphalt mastic is smaller than that of LS asphalt mastic. The $G^{*}$ value in asphalt mastic is positively correlated with its deformation resistance, indicating that SS asphalt mastic has the best high-temperature stability among the four types of asphalt mastics. As the temperature increases, the $G^{*}$ value of solid-waste-filler asphalt mastic gradually decreases. This is mainly because the mobility of the molecular functional groups of asphalt mastic increases with the increasing temperature, leading to increased repeated shear deformation at high temperatures and the formation of rutting. However, a higher modulus may be detrimental to the fatigue performance, which is related to the interaction between asphalt and solid waste materials. As shown in Figure 7b, the difference in δ between solid-waste filler and LS asphalt mastic is small, with RM being the largest and SS being the smallest, indicating that RM asphalt mastic has the most viscous component and SS asphalt mastic has the most elastic component. The solid-waste-filler asphalt mastic δ change rule was consistent. With the increase in temperature, the δ gradually increased, that is, the solid-waste-filler asphalt mastic transitioned from elastic to viscous, and the viscoelastic response tended to be viscous-dominant.

### 3.3. LAS Analysis

**Figure 8:**
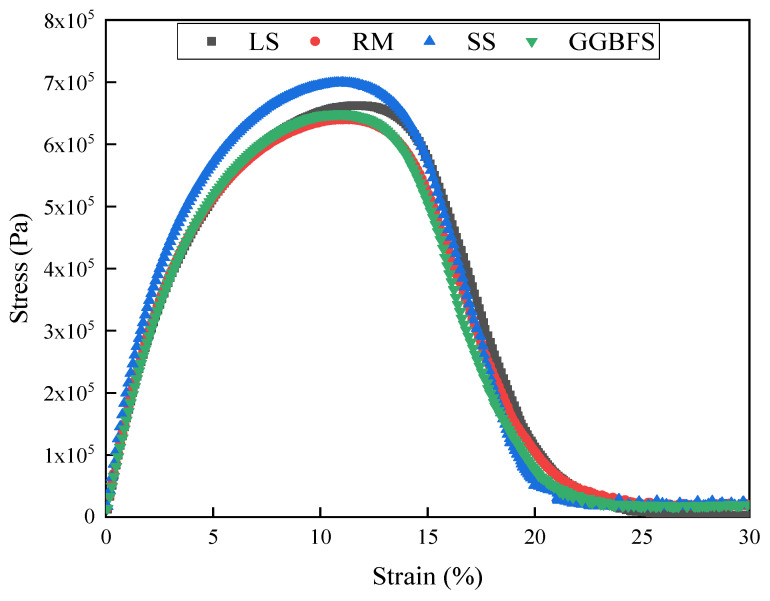
Stress–strain curve for solid-waste-filler asphalt mastic.

The stress–strain curve of the solid-waste-filler asphalt mastic in the LAS test is shown in Figure 8. The stress–strain curve of the solid-waste-filler asphalt mastic exhibited a parabolic trend. When the strain was low, the fatigue properties of the asphalt mastic samples exhibited the same trend and mainly exhibited viscoelastic characteristics. Under small strain, the slope of the stress–strain curves of each mastic are almost the same, and the interaction between the filler type and the asphalt mastic is not significant [40]. As the shear strain increased, the shear stress of the solid-waste-filler asphalt mastic exhibited an inflection point. In the strain amplitude sweep, the solid waste asphalt mastic is subjected to a very large strain and reaches the damage zone, where the influence of the material is more pronounced [36,40]. The four asphalt mastic stress–strain curves show a tendency for the stress to peak and then decrease owing to damage. The SS asphalt mastic exhibited the highest-yield stress, which was similar to that of RM and GGBFS. This is consistent with the maximum stiffness of SS asphalt mastic observed in the frequency and temperature sweeps. Since the stress–strain curves in the LAS test can only qualitatively determine the effect of the solid-waste filler type on the fatigue life of asphalt mastic, there is still a need for an in-depth analysis of other indicators in the LAS test.

**Figure 9:**
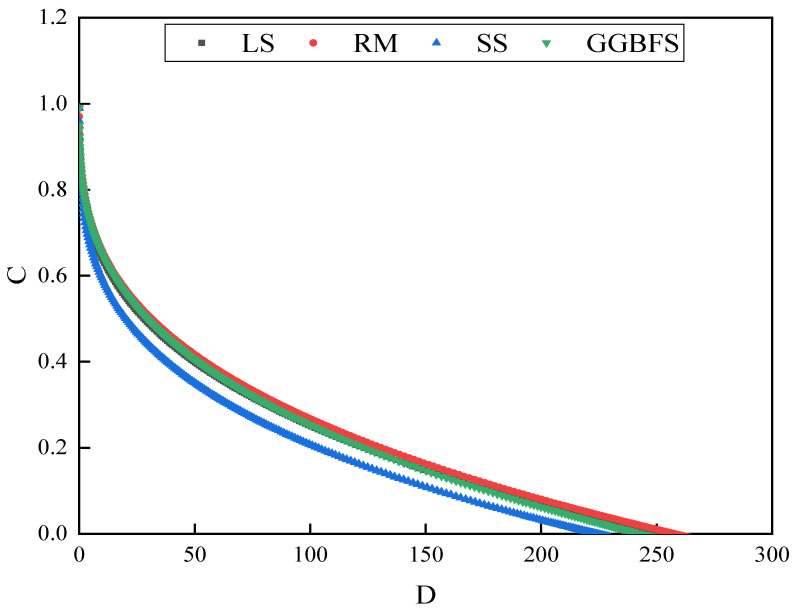
Fatigue damage curve for solid-waste-filler asphalt mastic.

Damage characteristic curves are basic features that represent the damage evolution process and predict the fatigue life of materials subjected to damage during loading [40]. The relationship between the fatigue damage characteristic curves in the LAS tests is shown in Figure 9. C = 1 indicates that the asphalt mastic did not cause any damage and possessed the highest integrity, whereas C = 0 indicates that the material underwent complete damage. When the cumulative damage parameter D is given, the larger the C, the more the material resists damage [41]. As shown in Figure 8, the trends of the damage curves of the solid-waste-filler asphalt mastic samples are similar, and the slope of the fatigue damage curve shows the rate of decrease in the damage index of each asphalt specimen, that is, the damage rate. The RM asphalt mastic sustained the highest cumulative damage, the SS asphalt mastic sustained the lowest cumulative damage when the same integrity index C was achieved, and the fatigue damage curves for the LS and GGBFS largely overlapped. The RM asphalt mastic exhibited the best fatigue life performance at the same temperature among the four asphalt mastics.

**Figure 10:**
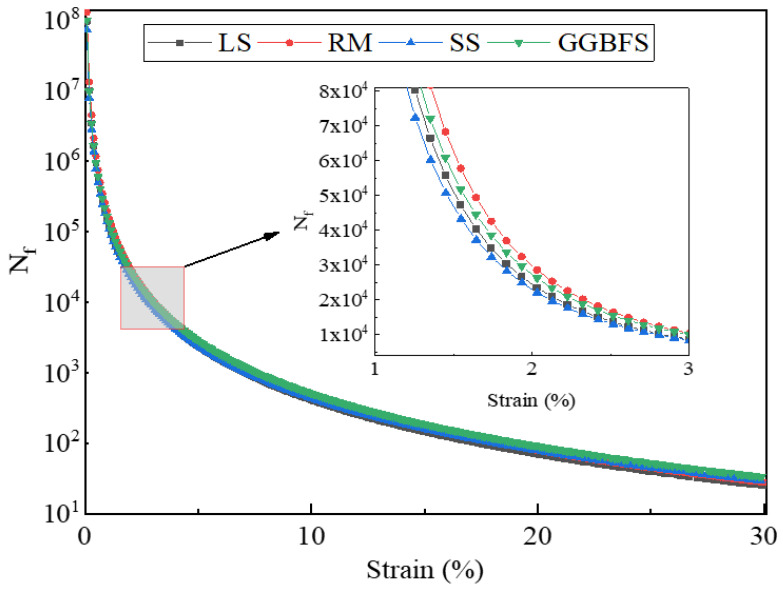
Fatigue life of solid-waste-filler asphalt mastic with strain.

The calculated fatigue life of the solid-waste-filler asphalt mastic with respect to the strain is shown in Figure 10. The fatigue life of the solid-waste-filler asphalt mastic was ranked as follows: RM > GGBFS > LS > SS. The fatigue life of the RM asphalt mastic reaches 16,470 and 2750 cycles at a 2.5% and 5% strain amplitude, respectively. The fatigue life of asphalt mastic using the SS filler always shows the lowest value, which is related to the high stiffness and brittleness of SS asphalt mastic. The higher the stiffness of the material, the smaller its strain response [40]. It can be clearly stated that compared to a low-strain amplitude, the fatigue life under a high-strain amplitude decreases more significantly. The solid-waste filler is filled in the asphalt, a filler–asphalt interface is generated inside the mastic, and the crack extension path is longer, which can improve the fatigue damage life of the mastic. However, solid-waste filler filling has a negative effect. The hardening of the asphalt mastic by the solid-waste filler increased the sensitivity of the fatigue life to strain, resulting in the lower fatigue life of the mastic under higher strain conditions. Therefore, in the LAS test, the positive effect of particle filling on the fatigue life, along with its negative effect on the corresponding sensitivity level, determined the fatigue performance of the asphalt mastic. At small strains, the enhancement of fatigue life by the particle filling of the filler is dominant, whereas as the loading strain increases, the greater strain sensitivity due to the hardening effect of the particle filler gradually dominates, leading to a more rapid decrease in fatigue life [14]. The fatigue life of asphalt mastic is related to the type of filler and structural form of the pavement [42].

### 3.4. MSCR Analysis

**Figure 11:**
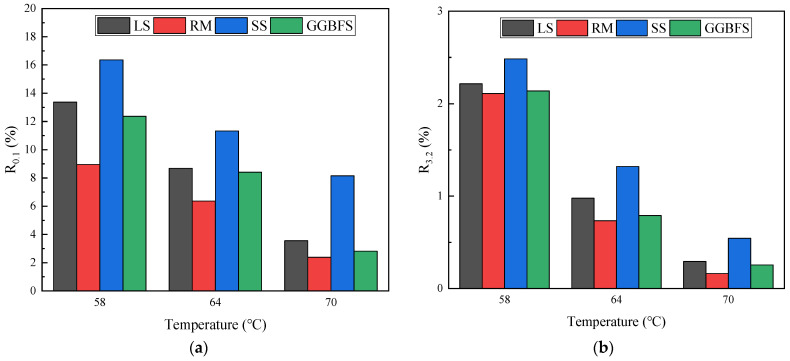
Percent recovery of solid-waste-filler asphalt mastic: (**a**) 0.1 kPa; (**b**) 3.2 kPa.

The improvement in the hardening degree and anti-rutting performance of asphalt mastic can be attributed to the increase in the percent recovery (*R*) and the decrease in nonrecoverable creep compliance ($J_{nr}$). In this study, three experimental temperatures of 58, 64, and 70 °C were selected to conduct an MSCR test on samples of short-term aged solid-waste-filler asphalt mastic to evaluate the creep recovery characteristics of the mastic. The *R* values of the solid-waste-filler asphalt mastic at 0.1 kPa are shown in Figure 11a. The results showed that the *R* values of the mastic materials gradually decreased with the increasing temperature. An increase in temperature changed the viscoelastic ratio and decreased the elastic composition and deformation recovery of the asphalt mastic. At the same temperature and stress level, the *R* values of the SS asphalt mastic were higher than those of the LS asphalt mastic, whereas those of the RM and GGBFS asphalt mastics were lower than those of the LS asphalt mastic. The R values of the SS, GGBFS, and RM asphalt mastic were 130.45, 96.85, and 73.28% of that of LS asphalt mastic at the 64 °C temperature level.

Increasing both the loading stress and test temperature reduced the elastic recovery rate properties of the asphalt, resulting in the more plastic residual strain and reduced high-temperature deformation resistance of the asphalt mastic; the higher the temperature, the greater the sensitivity of the asphalt mastic strain to temperature. The *R* values of the solid-waste-filler asphalt mastic at a stress level of 3.2 kPa are shown in Figure 11b. The creep recovery of solid-waste-filler asphalt mastic had similar trends at stress levels of 0.1 kPa and 3.2 kPa, but the absolute value of the recovery was significantly lower at higher stress levels. At the 64 °C temperature, SS, GGBFS, and RM asphalt mastics were 135.07, 80.90, and 75.15% of LS asphalt mastic. Comparing the solid-waste-filler asphalt mastic stress of 0.1 kPa and 3.2 kPa, the *R* values of asphalt mastic at 3.2 kPa were also lower because the higher the stress level, the higher the tire pressure on the actual pavement, and the greater the rut depth. The SS asphalt mastic exhibited the largest *R* value, indicating that it had the largest elastic component when subjected to the creep recovery loading sequence. 

**Figure 12:**
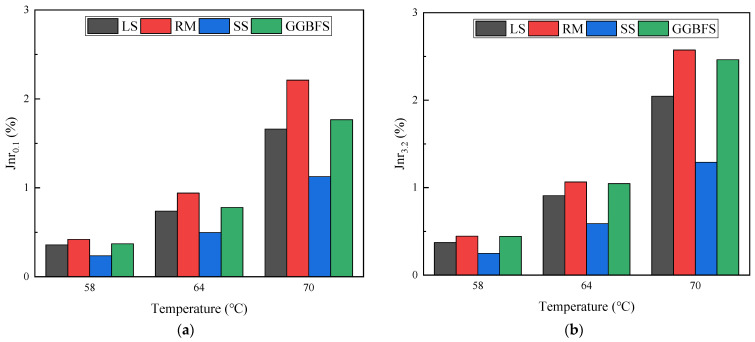
Nonrecoverable creep compliance of solid-waste-filler asphalt mastic: (**a**) 0.1 kPa; (**b**) 3.2 kPa.

The nonrecoverable creep compliance ($J_{nr}$) at a stress of 0.1 kPa is shown in Figure 12a. The smaller the $J_{nr}$ value of the asphalt mastic, the smaller the irrecoverable strain produced under stress, and the stronger the asphalt mastic’s ability to resist deformation. At a 64 °C temperature, SS, GGBFS, and RM asphalt mastic were 67.35, 105.60, and 127.57% of the $J_{nr}$ values of LS asphalt mastic. The $J_{nr}$ values of the solid-waste-filler asphalt mastic increased with the increasing temperature.

The $J_{nr}$ values at the stress of 3.2 kPa are shown in Figure 12b. At 64 °C, SS, GGBFS, and RM asphalt mastic were 64.81, 115.13, and 117.26% of the $J_{nr}$ values of LS asphalt mastic. For the same type of solid-waste-filler asphalt mastic, increasing the loading stress at the same temperature increased the $J_{nr}$ value. As the temperature increased, the elastic recovery stability of the mastic decreased, the stress sensitivity of the nonrecoverable permanent deformation increased, and the nonlinear viscoelastic behavior of the asphalt mastic was more pronounced at higher temperatures.

### 3.5. SFE Analysis

**Figure 13:**
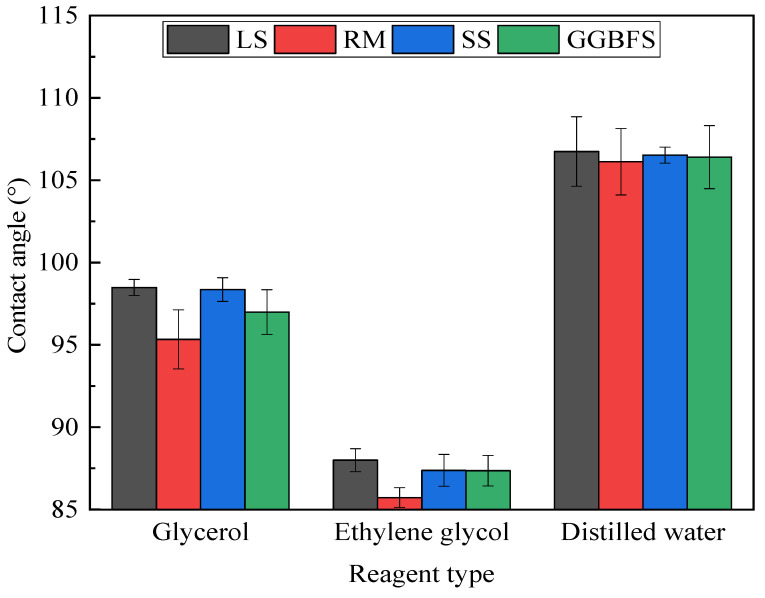
Contact angle of solid-waste-filler asphalt mastic.

The SFE of asphalt and solid-waste fillers are closely related to the filler particle size, surface structure, and surface chemistry [43]. In the SFE test, the contact angle on the surface of the asphalt mastic was measured with different probe liquids, and the results of the determination of the solid-waste-filler asphalt mastic contact angle are shown in Figure 13. The coefficient of variation (CV) of the test results ranged from 0.48% to 2.11%, indicating the suitable repeatability of the SFE test. The contact angles between the three liquids and the surface of the solid-waste-filler asphalt mastic differed. The asphalt mastic exhibited the largest contact angle with distilled water, followed by glycerol and ethylene glycol. In general, the larger the contact angle, the worse the wettability of the asphalt mastic with a particular liquid. This indicates that ethylene glycol is more likely to wet the asphalt mastic and that distilled water has the worst wetting properties, regardless of the type of asphalt mastic.

Through the contact angle test, the surface energy parameters of each type of solid-waste-filler asphalt mastic were calculated as shown in Table 4.

As shown in Table 4, based on the fitting equations, each type of asphalt mastic exhibited a suitable fit, which was greater than 0.98. The dispersive component of the SFE parameter of the solid-waste-filler asphalt mastic was larger than the polar component for the largest share of the surface energy composition, indicating that van der Waals forces contributed the most to the SFE of the asphalt mastic [44]. Owing to the different polar characteristics of solid-waste fillers, they interact differently with asphalt, thus changing its surface structure [45]. The LS, RM, SS, and GGBFS have different effects on the SFE components of asphalt mastics. The dispersion components were ordered as follows: LS > GGBFS > SS > RM, whereas the polar components were ordered in opposite directions. The type of solid-waste filler affected the surface activity of the asphalt mastic during the three liquid-wetting processes.

In asphalt mastics, the surfaces of the filler particles and asphalt in the hydrocarbon macromolecules come into contact, resulting in adsorption. Therefore, the surface characteristics of the filler have a certain degree of influence on the performance of the mastic. The SFE of LS asphalt mastic was the largest, indicating that LS had a higher activity after being wetted by the liquid. LS contains the most CaO, which acts as an alkaline oxide that enhances the adhesion, water stability, and spalling resistance of asphalt [46]. The SFE of RM, SS, and GGBFS were less than that of the conventional LS asphalt mastic. The SFE of the SS asphalt mastic is better than that of the RM and GGBFS asphalt mastics, possibly because the complex surface texture of the steel slag may lead to an increase in the surface coating of the aggregate with asphalt mastic, which may enhance the spalling resistance of the asphalt mastic [47]. Among the four fillers, because GGBFS contains the most SiO_2_, SiO_2_ leads to the increased hydrophilicity of the asphalt mastic, which makes it easier for the asphalt mastic to adsorb water, thus decreasing its SFE to asphalt mastic. Simultaneously, SiO_2_ affects the electrochemical reaction of the asphalt mastic, resulting in low SFE. The surface energy of RM is the smallest, which may be because RM contains more SiO_2_ and more Na_2_O among the four fillers, leading to the risk of water damage at the bonding interface between the red mud and asphalt owing to the chemical bonds formed between Na_2_O and asphalt, which are easily dissolved when exposed to water [48]. The difference in SFE between the solid-waste-filler asphalt mastic and the traditional LS asphalt mastic is not particularly obvious, indicating that the solid-waste-filler asphalt mastic can be used as an alternative filler from the perspective of SFE.

## 4. Conclusions

In this study, to investigate the feasibility of replacing LS with solid waste RM, SS, and GGBFS as fillers, the viscoelastic, fatigue, high-temperature, and wetting of asphalt mastics with solid-waste fillers were analyzed based on rheology and SFE theory. The main conclusions are as follows:

(1) Solid-waste fillers vary in shape and surface topography. The LS powder has an angular form with no visible voids. The single RM particle size was small, with some agglomerated particles. The SS powder has a fluffy structure and rough surface. GGBFS appeared irregularly granular with sharp edges.
(2) The SS asphalt mastic had the highest complex modulus and percent recovery with the best high-temperature rutting performance, elastic response, and permanent deformation ability, followed by the LS, GGBFS, and RM asphalt mastics. The viscoelastic response of solid-waste-filler asphalt mastic tends from viscosity to elasticity with an increasing loading frequency or decreasing temperature.
(3) The use of RM and GGBFS instead of LS improved the fatigue resistance of asphalt mastics. The particle filling and hardening effects of solid-waste fillers on asphalt affect the fatigue life of asphalt mastic.
(4) The dispersion component of the SFE parameter for the solid-waste-filler asphalt mastic was significantly larger than that of the polarity component. Solid-waste fillers have different chemical compositions and micro-morphologies, resulting in differences in the SFE of solid-waste-filler asphalt.

Based on these results, SS, GGBFS, and RM could potentially replace LS. However, the low-temperature performance, anti-aging performance, micro mechanism, crack resistance, and environmental impact of SS, GGBFS, and RM asphalt mastics will be verified in the subsequent research.

## Figures and Tables

**Table 1:** Properties of base asphalt binder.

Properties	Test Value	Specification	Method
Penetration (100 g, 5 s, 25 °C)/0.1 mm	67.9	60–80	T0604-2011
Softening point/°C	48.7	≥45	T0605-2011
Ductility (15 °C)/cm	>150	≥100	T0606-2011
Wax content/wt.%	1.2	≤2.2	T0615-2011
Flash point (COC)/°C	285	≥260	T0611-2011

**Table 2:** Surface energy parameters of test reagents.

Test Reagents	Surface Energy Parameters (mJ/m^2^)		
	$\gamma_{l}^{d}$	$\gamma_{l}^{P}$	$\gamma_{l}$
Ethylene glycol	29.0	19	48
Distilled water	21.8	51	72.8
Glycerol	34	30	64

**Table 3:** Chemical composition (wt.%) of the fillers.

Chemical Type	Materials Weight (wt.%)			
	LS	RM	SS	GGBFS
Na_2_O	0.175	8.141	0.454	0.505
MgO	0.876	0.118	4.337	9.810
Al_2_O_3_	1.727	26.372	6.660	17.196
SiO_2_	3.761	17.009	21.712	31.476
P_2_O_5_	0.023	0.387	0.418	0.016
SO_3_	0.056	0.650	0.526	2.142
Cl	0.026	0.066	0.049	0.049
K_2_O	0.343	0.373	0.151	0.354
CaO	91.803	1.442	29.638	35.921
TiO_2_	0.094	4.260	0.517	1.524
MnO	0.043	0.064	3.101	0.405
Fe_2_O_3_	1.023	40.245	30.454	0.295
SrO	0.050	0.011	0.038	0.043
F	-	0.561	-	0.222
Cr_2_O_3_	-	0.101	1.786	-
CuO	-	0.012	0.018	0.004
ZnO	-	0.007	0.021	-
Ga_2_O_3_	-	0.010	-	-
As_2_O_3_	-	0.010	-	-
Y_2_O_3_	-	0.007	-	0.006
ZrO_2_	-	0.144	0.016	0.034
Nb_2_O_5_	-	0.009	0.005	-
V_2_O_5_	-	-	0.100	-

**Table 4:** Surface energy of solid-waste-filler asphalt mastic.

Type	Fit Equation	*R^2^*	Surface Energy Parameters (mJ/m^2^)		
			$\gamma_{a}^{P}$ ^1^	$\gamma_{a}^{d}$ ^2^	$\gamma_{a}{\;}^{3}$
LS	y = 1.0355x + 3.9753	0.9951	1.07	15.80	16.87
RM	y = 1.2424x + 3.5639	0.9897	1.54	12.70	14.24
SS	y = 1.1856x + 3.7041	0.9999	1.41	13.72	15.13
GGBFS	y = 1.2319x + 3.6046	0.9807	1.52	12.99	14.51

^1^ Surface energy polar component; ^2^ Surface energy nonpolar dispersion component; ^3^ Total surface energy.

## Data Availability

Data available on request due to privacy.
